# Allelopathic effects of leaf extracts of *Eucalyptus camaldulensis* Dehnh. on morphological, physiological, and yield traits of Ethiopian wheat (*Triticum durum* L.) cultivars

**DOI:** 10.1186/s12870-024-05832-9

**Published:** 2024-11-28

**Authors:** Animut Mekuriaw Andualem, Mersha Wubie Aragaw, Abiyu Enyew Molla, Zelalem Getnet Tarekegn, Getinet Masresha Kassa

**Affiliations:** 1 Department of Biology, College of Science, Bahir Dar University, P.O.Box 79, Bahir Dar, Ethiopia; 2 Department of Biology, College of Natural and Computational Sciences, Debark University, P.O.Box 90, Gondar, Ethiopia; 3 Department of Biology, College of Natural and Computational Sciences, University of Gondar, P.O.Box 196, Gondar, Ethiopia

**Keywords:** Allelochemicals shoot length, Chlorophyll content, Dry weight, Fresh weight, Yield

## Abstract

Allelochemicals released into the soil from the leaves of eucalyptus species affect the growth and physiology of various crops. This study aimed to evaluate the allelopathic effects of aqueous and methanolic leaf extracts from *Eucalyptus camaldulensis* on three Ethiopian wheat cultivars (Assasa, Mukiye and Ude) of *Triticum durum* L. It was conducted as a pot experiment, and it utilized four concentrations of the extracts (Control (0%), 10%, 15%, and 20%) in a completely randomized design with three replicates. Results indicated that both extracts inhibited plant growth, biomass, and yield, with the methanolic extract showing stronger inhibitory effects. For instance, a concentration of 20% methanolic leaf extracts decreased chlorophyll fluorescence in the Assasa, Ude, and Mukiye cultivars by 53.97%, 36.36%, and 36.51%, respectively. The growth of both shoots and roots in Assasa, Ude, and Mukiye was significantly reduced at higher concentrations. Increasing concentrations of the extracts led to greater reductions in seedling traits and overall crop yield, with significant impacts observed (p ≤ 0.05). The findings suggest that eucalyptus should not be planted on agricultural land due to its negative impact on crop productivity.

## Introduction

Wheat (*Triticum durum* L, 2n = 4x = 28 chromosomes, AABB) belongs to the family Poaceae and the genus Triticum [1]. It has an annual average yield of 3.39 metric tons ha-1 [2] globally and 4 tons ha-1 with the overall production of 6.7 million tons under rain-fed agriculture in Ethiopia [3]. It is one of the most significant food crops in the country and its demand for the country has been growing over the years due to quick population growth and urbanization with necessitated alteration in meals preferences [3]. Wheat production in Ethiopia suffers from diseases, soil acidity, declining soil fertility, terminal moisture stress, and climate change [4]. Understanding the specific production constraints is essential for maximizing the production of local wheat cultivars. Allelopathy due to Eucalyptus tree is one of the root causes for the reduction of the crop productiveness in Ethiopia as the demand for the trees has become increased year to year.

Allelopathy refers to a phenomenon wherein chemical substances released by living or dead plant materials hinder the growth of other plants [5, 6]. These substances can be actively discharged through plant exudation [7] or generated passively during the decomposition of plant residues [8]. The chemicals produced by different plants are commonly referred to as allelochemicals, and they pose a significant challenge to wheat production by reducing productivity through competition [9]. Farmers in Ethiopia grow eucalyptus trees for different purposes within their farm vicinity. In this regard, a lot of farmers have additionally transformed their farmlands to eucalypt and assorted their profits [10]. These plant species produce chemical substances which intervene with different flora and have an effect on seedling growth. Allelochemicals produced from eucalyptus negatively affect agricultural productivity [11] and chemical compounds produced from *E. camaldulensis* have inhibitory outcomes on the seedling growth of some crops [12]. Wheat crops are amongst the most affected plant life by means of allelochemicals launched by using eucalyptus trees.

*E. camaldulensis* is categorized under the Myrtaceae family and the genus Eucalyptus. Even though Eucalyptus is not native to Ethiopia, it has been cultivated for the last century [13]. Eucalyptus trees take up farmland that could be utilized for growing food crops and adversely impact indigenous plant species, including crops [14]. A number of studies [15–18] have showed that *E. camaldulensis* restricts the growth of various agricultural crops such as maize, barely, bean, sorghum, millet, and tomato. Several earlier investigations have also indicated that aqueous solutions derived from Eucalyptus trees pose a hazard to seedling development [19–22]. Nevertheless, no comparative study has yet examined the impact of both aqueous and methanolic leaf extracts of *Eucalyptus camaldulensis* across a broad range of wheat crop features. This experimental research aims to internalize the effects of these extracts. Therefore, the current study seeks to assess the influence of both aqueous and methanolic leaf extracts of *E. camaldulensis* on the growth of selected wheat cultivars.

## Materials and methods

### Description of the experimental site

The experiment was conducted at the Botany Laboratory situated in the University of Gondar (12°35’11.7” N and 37°26’27” E, 2148 m above sea level). The yearly mean temperature ranges between 27 °C and 16 °C for maximum and minimum, respectively, while the mean precipitation and relative humidity are around 1161 mm and 56%, respectively. In the experimental timeframe, which was from January to April, the relative humidity was approximately 50.5%, and no precipitation was recorded while the maximum and minimum temperatures were 29.1 °C and 18 °C, respectively. The wheat plants require 120–240 days to grow, and all three cultivars have a similar growth rate.

### Collection of experimental materials

The seeds of the three wheat cultivars (namely: Assasa, Mukiye, and Ude) were obscured from Gondar Agricultural Research Center, Ethiopia. The foliage of *E. camaldulensis* was gathered in the vicinity of University of Gondar and transported to the laboratory.

### Experimental soil sampling and analysis

Prior to sowing the seeds of the cultivars, soil specimens were collected by an augur at random from the experimental field at a depth of 0–20 cm. The soil employed for the study was categorized as sandy loam in texture and had a pH of 7.21. The specimens were thoroughly blended and tested utilizing the procedure described by Shumet et al. [23]. The outcome of the experimental soil analysis is shown in Table 1.

Afterwards, the seeds of the three wheat cultivars (*Triticum durum* L.) were sanitized with 80% ethanol for approximately 15 min, immersed in purified water, and subsequently planted in plastic containers (25 cm in width × 26 cm in height) filled with 4 kg of soil. The process of gathering information began after a fortnight from the onset of seed sprouting. Following another fourteen days, the young plants in every container were reduced to three per pot.

**Table 1:** The physical and chemical properties of the experimental soil

Properties	Particle distribution	Units	Values
Physical properties	Clay	%	62.58
	Silt	%	22.67
	Sand	%	14.75
	Textural classification	Sandy loam soil	
Chemical properties	Moisture content	%	30.4
	pH		7.22
	EC	mscm^− 1^	0.689

### Preparation of both aqueous and methanolic leaf extracts of *E. camaldulensis*

The sample leaves were first cleaned meticulously using distilled water. The cleaned leaves allowed continuing to be at room temperature for drying carefully. After whole drying, leaves grinded by means of the use of dry electric powered grinder (model number, SZJ-831). Two types of extracts were prepared: aqueous and methanolic. For both extracts, a concentrated solution was created by blending 100 g of leaf extract with 1000 ml (1:10) of solvents [24]. The solvents used were distilled water for the aqueous extract and methanol for the methanolic extract. The amalgamated extracts were agitated for 72 h in a shaker and stored in a refrigerator (4^o^c) for 48 h. Following this, the solution was sifted using Whatman No.1 filter paper. The residue was disposed of, and the filtrate was utilized for subsequent testing, following to vaporizing the solvents. To remove the solvent, the watery infusion was subjected to a temperature of 100 ^o^C, while the methanol extract was put in a water bath at 70^o^C to eliminate methanol until the solvents were completely vaporized [25]. The resulting concentrated extracts were considered the 100% (w/v) standard aqueous and methanolic leaf extracts. From these standard stock solutions, working concentrations of 10%, 15%, and 20% (w/v) were prepared by mixing with distilled water, following the method of Akram et al. [26]. The process involved weighing 10 g, 15 g, and 20 g of both aqueous and methanolic leaf extracts from the original concentration individually, and subsequently adding 100 ml of distilled water to each of the 10 g, 15 g, and 20 g extracts. The extract values were calculated following the formula of Adelanwa et al. [24] as follows;

$$\text { Extract value }=\frac{\text { Weight of the extract }(\mathrm{gm})}{\text { Weight of powdered of leaf }(\mathrm{gm})} \times 100 \%$$

### Treatments and experimental design

A pot experiment was carried out using a completely randomized design (CRD) comprising of four treatments and three replications. In this experiment, four aqueous extract treatments were applied to the seedlings of wheat cultivars (Assasa, Mukiye, and Ude). The treatments were as follows: T_0_ (control) using only tap water, T_1_ = 10% concentration of Eucalyptus aqueous extract, T_2_ = 15% concentration of Eucalyptus aqueous extract, and T_3_ = 20% concentration of Eucalyptus aqueous extract. Similarly, four methanolic extract treatments were applied to other seedlings of the same cultivars. These treatments include; T_0_ (control) using tap water, T_1_ = 10% concentration of Eucalyptus methanolic extract, T_2_ = 15% concentration of Eucalyptus methanolic extract, and T_3_ = 20% concentration of Eucalyptus methanolic extract. A total of 72 pots (36 for the aqueous experiment and 36 for the methanolic experiment) were arranged in three rows, with each pot containing 4 kg of soil. Ten wheat seeds were sown in each pot for the experiment [27]. All pots were given the same amount of water, 450 ml, in accordance with the crop’s water needs. The seedlings were watered daily, and the treatments were given every five days. The tap water’s electrical conductivity used in the study had an average of 0.0066 dS/m, and the average pH was determined to be 6.98.

### Data collection and measurements

#### Phytochemical screening of *E. camaldulensis* leaf extracts

The presence or absence of secondary metabolites in the leaf extracts of *E. camaldulensis* was analyzed using the method described by Adelanwa et al. [24].

#### Morphological traits

Shoot length (SL), root length (RL), stem thickness (ST), number of leaves (No.L), and leaf area (LA) were measured in each treatment under both leaf extract conditions. The shoot length and root length were measured using graduated rulers and the mean value was taken as the length in each treatment. The seedlings were exposed to aqueous and methanolic leaf extracts at concentrations of 10%, 15%, and 20% for 30 days. Shoot length was measured at 0, 10, 20, and 30 days of exposure to evaluate the time-dependent effects. For both the aqueous and methanolic extracts, shoot length at day 0 was compared with the measurements taken on subsequent days (10, 20, and 30) to assess the effects of the extracts separately over time. Number of green leaves, (with more than 50% green part) was counted and the leaf area was measured using the formula of Otusunya et al. [28]. as follows;

$$\mathrm{LA}=0.5(\mathrm{~L} * \mathrm{~W})$$

Where L = length of leaf, W = maximum width.

The stem thickness per plant in each pot was measured by using a Digital Vernier hand caliper meter (AP-961) and the mean for stem thickness of the individual plants in the replicate was taken as the stem thickness.

#### Physiological and biochemical traits

It was carefully assessed what the physiological and biochemical characteristics were, including relative leaf water content (RLWC), chlorophyll fluorescence (CF), chlorophyll concentration, and carotenoid (Cn) content. The RLWC was calculated from sampled cultivar leaves in all treatments using the method of Lugojan and Ciulca [29] as follows;

$$\eqalign{& {\rm{ RLWC}}(\% ){\rm{ = }}{{{\rm{ FW - DW }}} \over {{\rm{ TW - DW }}}}{\rm{X100, }} \cr & {\rm{Where, FW = Fresh weight, DW = Dry weight, TW = Turgid weight}}{\rm{. }} \cr}$$

The portable Multi-Mode Chlorophyll Fluorometer (ADC Bio Scientific Ltd., England) was used to measure the chlorophyll fluorescence (Fv/Fm) in the morning from 10:00 to 11:00 AM. The youngest and most enlarged leaves of wheat cultivars were used to make the determination. As recommended by Bachheti et al. [30], the leaves were kept in the dark for 30 min before to measurements by covering them with a clip for darker adaption.

Fresh leaves weighing 0.5 g were obtained and homogenized in a tissue homogenizer with 10 ml of 80% acetone solvent to find the amount of chlorophyll. For 10 min, the homogenized sample was centrifuged at 5000 rpm. In the cubit test, 4.5 ml of the acetone solvent and around 0.5 ml of the sample were combined. Using a microprocessor UV-Visdouble-beam spectrophotometer, the solution mixture’s concentration of chlorophyll-a, chlorophyll-b, and carotenoids were examined. The formulas of Sumanta et al. [31] were used to quantify chlorophyll-a, chlorophyll-b, and carotenoids in an acetone extract.

Ch-a = 12.25A663.2 -279A646.8.

Ch-b = 21.5A646.8-5.1A663.2 and.

Cn= (1000A470-1.82Ca -85.02Cb)/198.

Where (A663.2) and (A646.8) represent absorbance values read at 663.2 and 646.8 nm wavelengths and A = Absorbance, Ch-a = Chlorophyll a, Ch-b = Chlorophyll b, Cn = Carotenoids.

#### Biomass and yield traits

##### Fresh and dry weight

At the end of the experiment the plants were uprooted by adding plenty of water to the pot. The shoots, yield pods and roots were separated by using scissor. The fresh weights of the shoot, root and yield were measured separately by using digital electrical balance. Then, the mean of fresh weight of the control and the treatments were recorded. The dry weight of the shoot root, and yield were measured after putting in an oven at 70^o^C. After drying, the shoot, root and yield were separated and weighed by using digital electric balance and they were summed up separately. Then, the mean of dry weight of the control and the treatments were recorded.

### Percent inhibition

Percent inhibition on the biomass and yield traits of the three wheat cultivars due to the effect of allelochemicals was computed following the formula of Enyew and Raja [32] as follows;

$$\text { Percent inhibition }=\frac{(\text { Control-Treatment })}{\text { Treatment }} \mathrm{X} 100$$

### Data analysis

All the collected data were analyzed using SPSS version 26 at 95% confidence interval (*p* ≤ 0.05). Analysis of variance (one way ANOVA) was used to test the significance of the main effects. All means of the controls were compared to the treatments for all significant parameters using least significant difference (LSD) test. The data for the aqueous extract were analyzed using one-way ANOVA, followed by LSD test to compare shoot length at different exposure times (0, 10, 20, and 30 days). For the methanolic extract, data were similarly analyzed using one-way ANOVA, followed by LSD to assess significant differences in shoot lengths across different exposure times. A significance level of *p* ≤ 0.05 was considered statistically significant. Two- way ANOVA was also used to evaluate the interaction effect of the cultivar type and allelopathic effect on different parameters. Correlation of many growth parameters and leaf extract concentration was calculated. The growth parameters were correlated by using Pearson correlation measure(r).

## Results and discussion

### Phytochemical screening of *E. camaldulensis* leaves

The investigation of phytochemicals in the aqueous and methanolic extracts of *E. camaldulensis* leaves revealed the presence of saponins, phenols, tannins, flavonoids, alkaloids, and steroids, as indicated in Table 2. This agrees with the previous reports [33, 34] on the methanolic leaf extracts of *E. camaldulensis*. However, contrary to the results of the present study, Ibrahim et al. [35] reported the absence of glycosides and anthraquinones in the aqueous leaf extracts of *E. camaldulensis*. The study further indicated that methanolic leaf extracts contained a higher concentration of allelochemicals compared to their aqueous counterparts. This could be attributed to the non-polarity of the allelochemicals which could be dissolved in methanol [36, 37]. The polarities of water and methanol differ, influencing their ability to dissolve secondary metabolites, and hence, polar solvents contain the highest amount of phytochemicals [38]. In contrast to the findings of this study, alkaloids and flavonoids were absent in the aqueous leaf extracts of Eucalyptus species [39]. This may be related to genetic variation, environmental factors, and differences in biosynthetic pathways within the genus.

**Table 2:** Phytochemical screening of *E. camaldulensis* leaf extracts

Phytochemicals	Aqueous extract	Methanolic extract
Saponins	+	+
Phenols	+	+
Tannins	+	+
Glycosides	-	+
Flavonoids	+	+
Anthraquinones	+	-
Alkaloids	+	+
Steroids	-	+

+ = presence of phytochemical; - = absence of phytochemical

### Effects on morphological traits

#### Shoot length

**Fig. 1:**
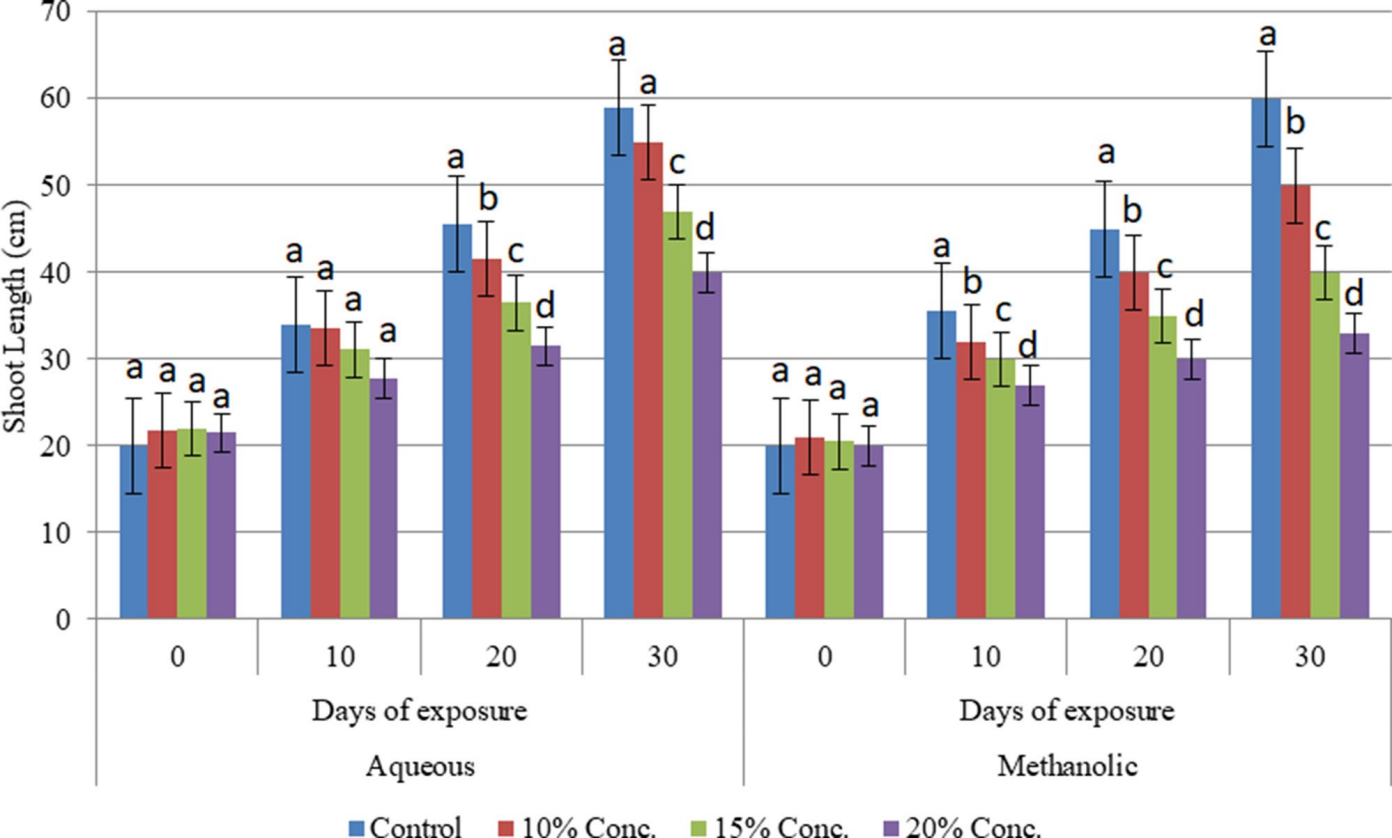
Effects of aqueous and methanolic leaf extracts on shoot length at different exposure days in Assasa cultivar tested separately over time. Bars with different letters show significant differences at *p* ≤ 0.05

**Fig. 2:**
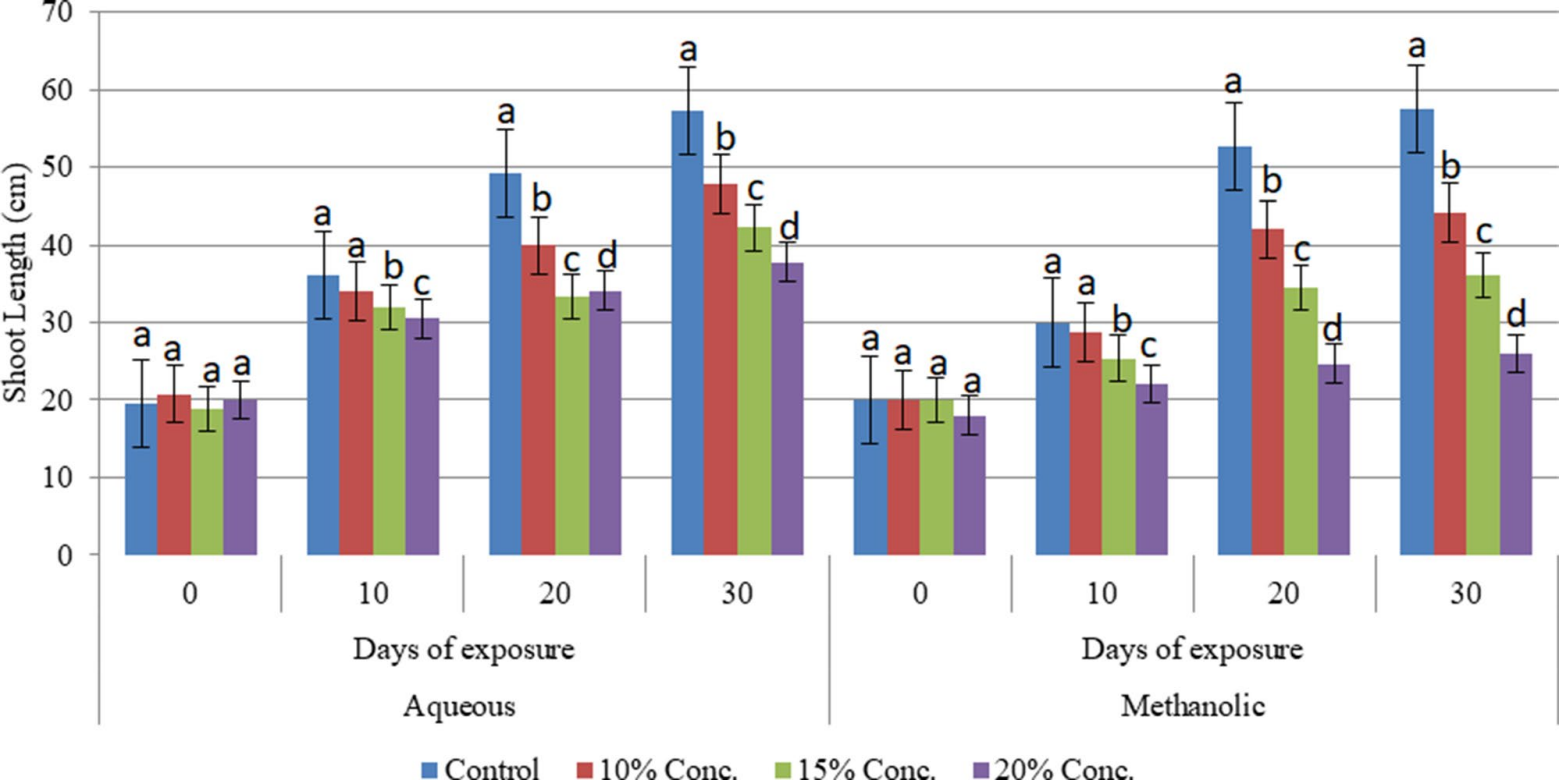
Effects of aqueous and methanolic leaf extracts on shoot length at different exposure days in Ude cultivar tested separately over time. Bars with different letters show significant differences at *p* ≤ 0.05

**Fig. 3:**
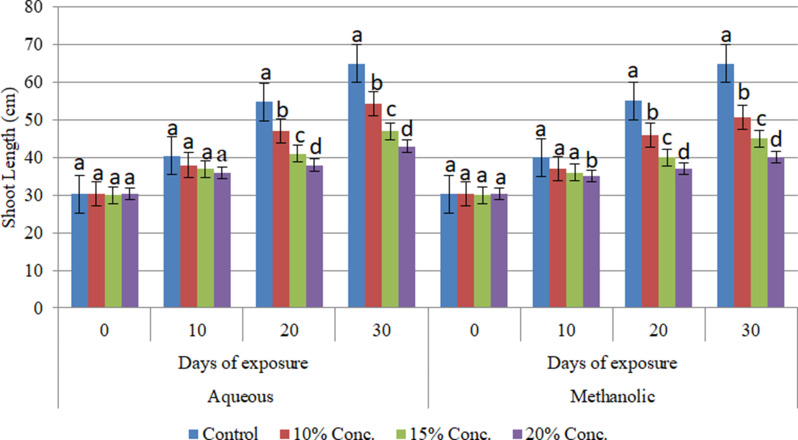
Effects of aqueous and methanolic leaf extracts on shoot length at different exposure days in Mukiye cultivar tested separately over time. Bars with different letters show significant differences at *p* ≤ 0.05

Alterations in the shoot growth of the cultivars were evaluated under different concentrations of aqueous and methanolic leaf extracts of *E. camaldulensis* after being subjected to stress for 30 days. The changes were analyzed at 10-day intervals until the end of the experiment. The study confirmed that the aqueous and methanolic leaf extracts of E. camaldulensis had a significant impact (at *P* ≤ 0.05 level) on the shoot length of the three wheat cultivars. It has been found that the shoot length of the Assasa, Ude and Mukiye cultivars was significantly reduced, when subjected to 15% and 20% concentrations of aqueous and methanolic leaf extracts of *E. camaldulensis* for 30 days compared to the control (Figs. 1, 2 and 3). The highest inhibition was recorded at higher concentrations of the methanolic leaf extracts, and the inhibitory effect of the extracts was found to be directly proportional to the concentration. This finding is consistent with previous studies [17, 40–42]. Other studies [17, 43, 44] on crops such as tomato, teff, barely, and maize have investigated the allelopathic effects of Eucalyptus species, revealing substantial inhibitory impacts of their extracts. Similarly, the allelochemicals in Eucalyptus leaf extracts have been shown to impede the shoot length of radish, onion, and tomato plants [45]. Allelopathic chemicals from weeds and flowering plants such as Bauhinia Species have also effect on the growth of wheat crops [46, 47]. The presence of phytochemicals in the leaf extracts likely contributes to this inhibitory effect on shoot length [48, 49]. This may be due to the phenolic compounds and volatile substances in Eucalyptus foliage, which can have detrimental effects on plant shoot growth [44, 49]. It may also be due to the reduced cell turgor pressure. Allelopathy could affect all physiological processes in the plant, including respiration, photosynthesis, chlorophyll formation, and plant water relationships [16, 50, 51].

#### Root length

The root length of all three cultivars was significantly affected by the application of both aqueous and methanolic leaf extracts on the seedlings (at *p* ≤ 0.05) when compared to the control group (Table 3). Though higher concentrations of both extracts resulted in allelopathic effects [17, 43], the inhibitory effect was greater with the methanolic leaf extract on the crops’ root length. This aligns with the findings of Dejam et al. [41], who reported that the methanolic leaf extract of eucalyptus exhibited a more pronounced inhibitory effect on the root length of eggplant varieties compared to the aqueous leaf extract. The aqueous extracts of *E. camaldulensis* has also effects on the growth of maize radicles [14, 52].

The findings showed that the stem thickness was significantly reduced (*p* ≤ 0.05) in Assasa, Ude, and Mukiye cultivars by 88.2%, 57.14%, and 76.92% respectively, when treated with a 20% concentration of *E. camaldulensis* leaf extract in water. Additionally, the stem thickness was also significantly reduced (*p* ≤ 0.05) in Assasa, Ude, and Mukiye cultivars by 88.89%, 64.29%, and 84.62% respectively, when treated with a higher concentration of *E. camaldulensis* leaf extract in methanol. It can be concluded that the inhibitory effect of the methanolic leaf extract was more pronounced on the stem thickness compared to the aqueous leaf extract.

#### The number of leaves and leaf area

The number of foliage declined significantly (*p* ≤ 0.05) as the concentration of the leaf extracts being analyzed increased (Table 3). This corresponds with the reports of Ziaebrahimi et al. [53] and Abou El-Ghit [54]. In a similar fashion, a noteworthy (*P* ≤ 0.05) decrease in leaf area was detected in the seedlings of all three types that were cultivated using the 20% treatment of both aqueous and methanolic leaf extracts obtained from *E. camaldulensis*.

The concentration of both leaf extracts under study resulted in a significant decrease (*p* ≤ 0.05) in the number of leaves (Table 3). This finding is consistent with the report of Ziaebrahimi et al. [53]. Dafaallah and El-Twom [14] also confirmed a significant reduction in the number of leaves in sorghum and maize seedlings due to the effects of *E. camaldulensis* leaf extracts. On the other hand, treatment with 20% aqueous and methanolic leaf extracts of *E. camaldulensis* caused a significant (*P* ≤ 0.05) reduction in leaf area of the three cultivars of seedlings. However, the methanolic leaf extracts of *E. camaldulensis* had a more pronounced allelopathic effect on leaf number and leaf area. The results agree with those reported by Djanaguiraman et al. [55] on cowpea plants, where the reduction of leaf number and leaf area was observed under the effect of allelochemicals. The allelochemicals released from Eucalyptus leaves may have stopped plant processes, which in turn could have affected many physiological processes in cowpea [55]. This may have directly or indirectly led to a decrease in the size and area of the leaf.

**Table 3:** Effects of *E. camaldulensis* leaf extracts on root length, stem thickness, number of leaves and leaf area

CV	Conc.	Aqueous leaf extract				Methanolic leaf extract			
		RL (cm)	ST (mm)	No.L/plant	LA (mm^2^)	RL (cm)	ST (mm)	No.L/plant	LA (mm^2^)
Assasa	Control	12.2 ± 0.3^a^	1.7 ± 0.1^a^	6.5 ± 0.0^a^	28 ± 2.0^a^	11.3 ± 0.5^a^	1.8 ± 0.1^a^	6.5 ± 0.5^a^	28.4 ± 1.4^a^
	10%	10.7 ± 0.6^ab^ (12.30)	1.7 ± 0.1^a^ (0.0)	5.7 ± 0.3^b^ (12.31)	24 ± 2.0^b^ (14.29)	9.70 ± 0.6^b^ (14.16)	1.3 ± 0.1^b^ (27.78)	5.3 ± 0.3^b^ (18.46)	22.6 ± 2.5^b^ (20.42)
	15%	10.3 ± 0.5 ^b^ (15.57)	1.3 ± 0.1^b^ (23.53)	4.8 ± 0.6^c^ (26.15)	16 ± 2.0^c^ (42.86)	8.70 ± 0.5^c^ (23.01)	0.6 ± 0.2^c^ (66.67)	4.3 ± 0.6^c^ (33.85)	15.0 ± 1.2^cd^ (47.18)
	20%	9.3 ± 0.6 ^cd^ (23.77)	0.2 ± 0.0^cd^ (88.2)	4.0 ± 0.5^d^ (38.46)	13 ± 4.0^gg^ (53.57)	8.00 ± 0.1^c^ (29.20)	0.2 ± 0.1^d^ (88.89)	3.6 ± 0.8^d^ (44.62)	10.5 ± 0.1^d^ (63.03)
Ude	Control	11.0 ± 0.0 ^a^	1.4 ± 0.1^a^	5.5 ± 0.5^a^	31 ± 5.0^a^	11.0 ± 0.4^a^	1.4 ± 0.3^a^	5.7 ± 0.3^a^	31.0 ± 1.8^a^
	10%	10.0 ± 0.0 ^b^ (9.09)	1.0 ± 0.1^bb^ (28.57)	4.8 ± 0.3^b^ (12.73)	18 ± 1.0^b^ (41.94)	10.0 ± 0.7^bd^ (9.09)	0.9 ± 0.5^b^ (35.71)	5.2 ± 0.6^aa^ (8.77)	17.4 ± 2.2^b^ (45.16)
	15%	8.3 ± 0.5^c^ (24.55)	0.7 ± 0.1^c^ (50)	4.5 ± 0.5^b^ (18.18)	15 ± 1.0^c^ (51.61)	8.20 ± 0.3^c^ (25.46)	0.7 ± 0.1^c^ (50)	4.5 ± 0.0^b^ (21.05)	12.9 ± 1.0^c^ (60.0)
	20%	7.3 ± 0.6^d^ (33.64)	0.6 ± 0.1^ee^ (57.14)	4.2 ± 0.3^c^ (23.64)	12 ± 1.0^d^ (61.29)	7.30 ± 0.6^d^ (33.64)	0.5 ± 0.4^d^ (64.29)	3.8 ± 0.3^c^ (33.33)	11.7 ± 0.0^cd^ (62.26)
Mukiye	Control	11.3 ± 0.6^a^	1.3 ± 0.4^a^	5.2 ± 0.3^a^	25 ± 3.0^a^	12.7 ± 0.2^a^	1.3 ± 0.1^a^	5.3 ± 0.3^a^	24.8 ± 2.7^a^
	10%	11.0 ± 0.0^a^ (2.65)	1.2 ± 0.1^a^ (7.69)	5.0 ± 0.0^a^ (3.85)	18 ± 1.0^b^ (28.00)	10.9 ± 0.1^b^ (14.17)	1.1 ± 0.5^b^ (15.39)	4.3 ± 0.2^b^ (18.87)	16.6 ± 2.1^b^ (33.07)
	15%	10.7 ± 0.6^a^ (5.31)	0.6 ± 0.3^b^ (53.85)	4.8 ± 0.8^a^ (7.69)	14 ± 0.0^c^ (44.00)	9.70 ± 0.6^c^ (23.62)	0.5 ± 0.1^cc^ (61.54)	4.1 ± 0.3^b^ (22.64)	12.8 ± 0.8^cd^ (48.39)
	20%	10.3 ± 0.6^bbd^ (8.85)	0.3 ± 0.1^b^ (76.92)	4.1 ± 0.7^bb^ (21.15)	13 ± 1.0^c^ (48.00)	9.30 ± 0.5^ff^ (27.64)	0.2 ± 0.1^d^ (84.62)	3.3 ± 0.2^cc^ (37.74)	11.4 ± 0.8^c^ (54.03)

The data presented shows the mean ± SD values, and the values in parentheses represent the percentage of inhibitory effects compared to the control. Different letters in the columns indicate significant differences according to the LSD test at a 0.05 probability level

### Effects on physiological traits

#### Relative leaf water content

The methanolic extract obtained from the leaves has a greater inhibitory impact on the relative water content of the leaves (Table 4). This finding is in line with previous reports [56, 57]. On the other hand, the aqueous leaf extracts showed a lesser inhibitory effect on RLWC in the three wheat cultivars. This could be due to the fact that the methanolic extract dissolved more phytotoxic allelochemicals than the aqueous extract. This is consistent with the findings of Ataollahi et al. [36], which suggest that organic solvents like methanol and ethyl acetate have greater phytotoxic activity than water-soluble allelochemicals.

#### Chlorophyll fluorescence

The findings showed that when a methanolic leaf extract was used at a concentration of 20%, it decreased chlorophyll fluorescence in the Assasa, Ude, and Mukiye cultivars by 53.97%, 36.36%, and 36.51%, respectively. However, an aqueous leaf extract at the same concentration had a lesser inhibitory effect on the Assasa, Ude, and Mukiye cultivars, with reductions of 32.26%, 7.55%, and 34.43%, respectively. The application of a 20% concentration had a significant impact on the chlorophyll fluorescence of the three cultivars when compared to the control. It is known that allelochemicals in plants can hinder the function of PS II [58].

**Table 4:** Effects of leaf extracts of *E. camaldulensis* on physiological traits

Cultivars	Conc.	Aqueous leaf extract		Methanolic leaf extract	
		RLWC (%)	CF (Fv/Fm)	RLWC (%)	CF (Fv/Fm)
Assasa	Control	78.0 ± 0.5^a^	0.62 ± 0.02^a^	78.5 ± 0.9^a^	0.63 ± 0.02^a^
	10%	69.0 ± 0.5^b^ (11.54)	0.55 ± 0.02^a^ (11.29)	64.5 ± 0.5^b^ (17.83)	0.51 ± 0.02^b^ (19.05)
	15%	63.0 ± 0.8^cc^ (19.23)	0.45 ± 0.04^bb^ (27.42)	55.3 ± 1.1^c^ (29.55)	0.38 ± 0.02^c^ (39.68)
	20%	60.0 ± 1.0^d^ (23.08)	0.42 ± 0.13^bcd^ (32.26)	44.5 ± 0.7^d^ (43.31)	0.29 ± 0.01^d^ (53.97)
Ude	Control	69.0 ± 1.0^a^	0.53 ± 0.03^a^	69 ± 1.0^a^	0.55 ± 0.03^a^
	10%	65.0 ± 1.0^b^ (5.80)	0.51 ± 0.02^b^ (3.77)	62.5 ± 0.5^b^ (10.15)	0.51 ± 0.01^a^ (7.27)
	15%	60.0 ± 1.0^c^ (13.04)	0.50 ± 0.05^cc^ (5.66)	55 ± 1.0^c^ (20.29)	0.47 ± 0.02^b^ (14.55)
	20%	55.0 ± 1.0^ddf^ (20.29)	0.49 ± 0.01^d^ (7.55)	41.2 ± 0.4^gg^ (40.29)	0.35 ± 0.04^cc^ (36.36)
Mukiye	Control	59.0 ± 0.1^a^	0.61 ± 0.02^a^	59.3 ± 0.4^a^	0.63 ± 0.02^a^
	10%	55.0 ± 0.6^bb^ (6.78)	0.48 ± 0.04^b^ (21.31)	49.9 ± 1.0^b^ (15.85)	0.48 ± 0.01^b^ (23.81)
	15%	48.0 ± 0.8^c^ (18.64)	0.43 ± 0.02^c^ (29.51)	42.3 ± 1.1^c^ (28.67)	0.41 ± 0.02^cc^ (34.92)
	20%	40.0 ± 0.6^def^ (32.20)	0.40 ± 0.01^ee^ (34.43)	37.3 ± 1.1^dd^ (37.10)	0.40 ± 0.01^c^ (36.51)

The data presented shows the mean ± SD values, and the values in parentheses represent the percentage of inhibitory effects compared to the control. Different letters in the columns indicate significant differences according to the LSD test at a 0.05 probability level

#### Chlorophylls and carotenoids content

**Fig. 4:**
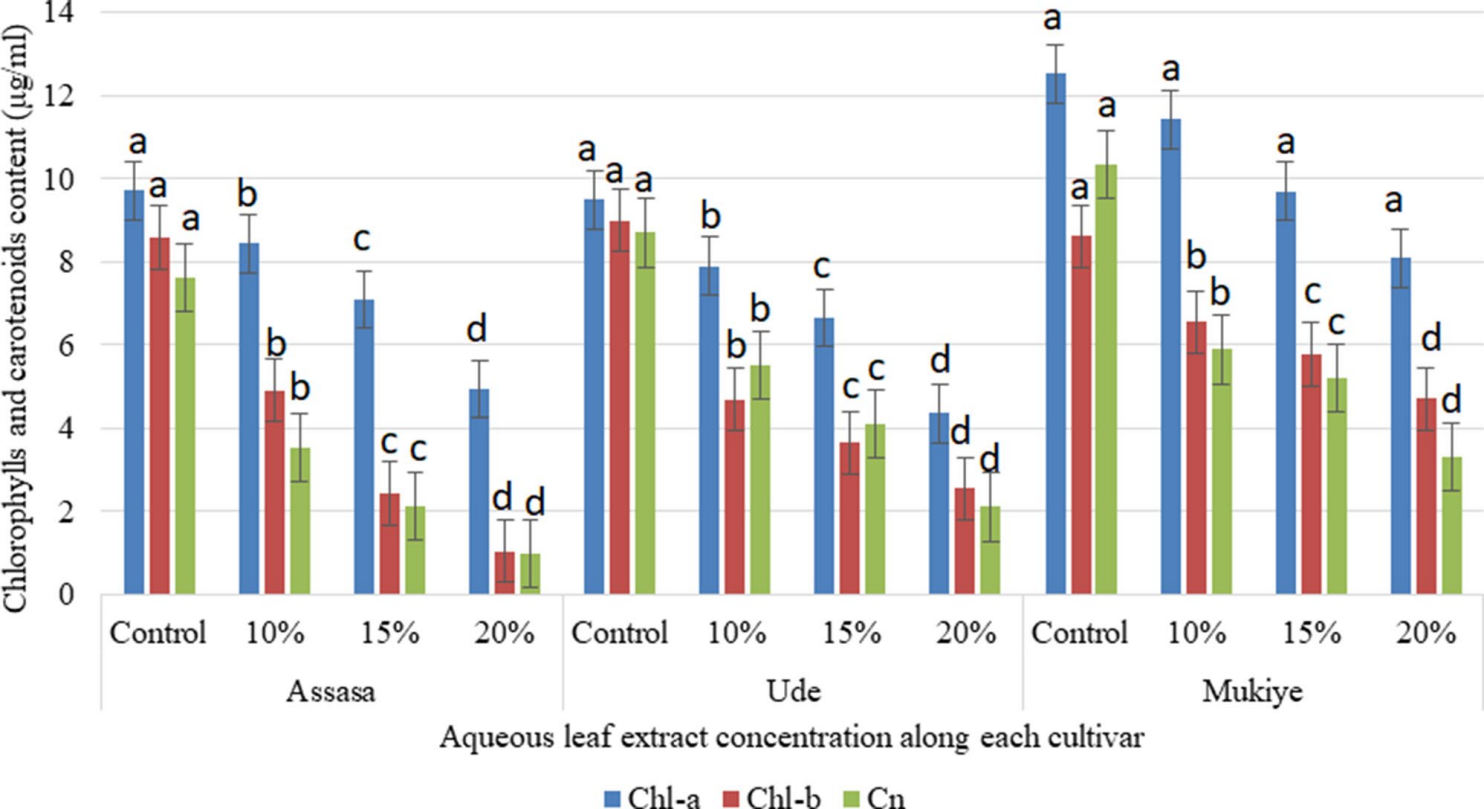
Effects of aqueous leaf extracts on chlorophylls a, b, and carotenoids of the three cultivars. Bars with different letters show significant differences at *p* ≤ 0.05

**Fig. 5:**
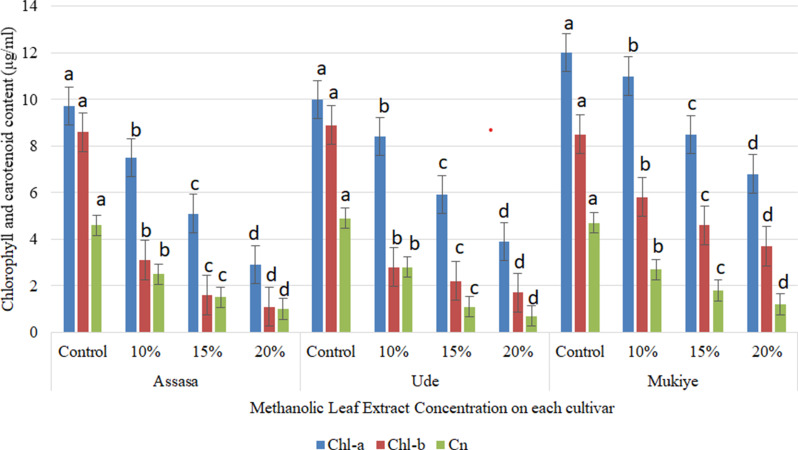
Effects of methanolic leaf extracts on chlorophylls a, b, and carotenoids of the three cultivars. Bars with different letters show significant differences at *p* ≤ 0.05

The contents of chlorophylls and carotenoids were considerably decreased in all treatments (Figs. 4 and 5). The methanolic leaf extracts exhibited stronger inhibitory effects on chlorophyll a in the Assasa cultivar, while a higher concentration of aqueous leaf extracts displayed greater inhibitory effects on chlorophyll b and carotenoids in the Assasa cultivar compared to the control. These findings align with the previous report [59]. The application of eucalyptus leaf extracts reduced the total chlorophyll content in maize and kidney beans [14, 44]. The varying degrees of chlorophyll reduction may be attributed to their differing susceptibility to stress [55], while the decline in chlorophyll content observed across all concentrations could also be due to the breakdown of chlorophyll pigments and the influence of diverse phytochemicals present in leaf leachates [60]. An increase in concentration resulted in a decline of chlorophyll content in *Oryza sativa* plants [61]. Eucalyptus plants’ aqueous and methanol leaf extracts have varying allelochemicals that possess the ability to cause allelopathic effects [62]. These compounds have been known to cause damage to the photosynthetic mechanism [63]. The leaf extracts of Eucalyptus that are volatile led to a marked reduction in photosynthetic pigments of *Amaranthus viridis* [64]. The eucalyptus leaf extract may reduce and slow down the biosynthesis of chlorophyll a and b [54].

Likewise, increased concentrations of both leaf extracts led to a considerable decrease in carotenoid content. Nevertheless, methanolic leaf extracts of *E. camaldulensis* showed higher inhibition on it in all cultivars. The inhibitory effects were particularly pronounced in the Ude cultivar at higher concentrations (20%) of the methanolic leaf extract. Reductions of carotenoid content due to the effects of aqueous leaf extracts of *E. camaldulensis* was also reported by El-Bakkosh et al. [65]. Previous studies also showed the allelopathic effects of eucalyptus species on the carotenoid contents of crops [54, 66].

### Percent inhibition

#### Shoot fresh weight (SFW)

**Fig. 6:**
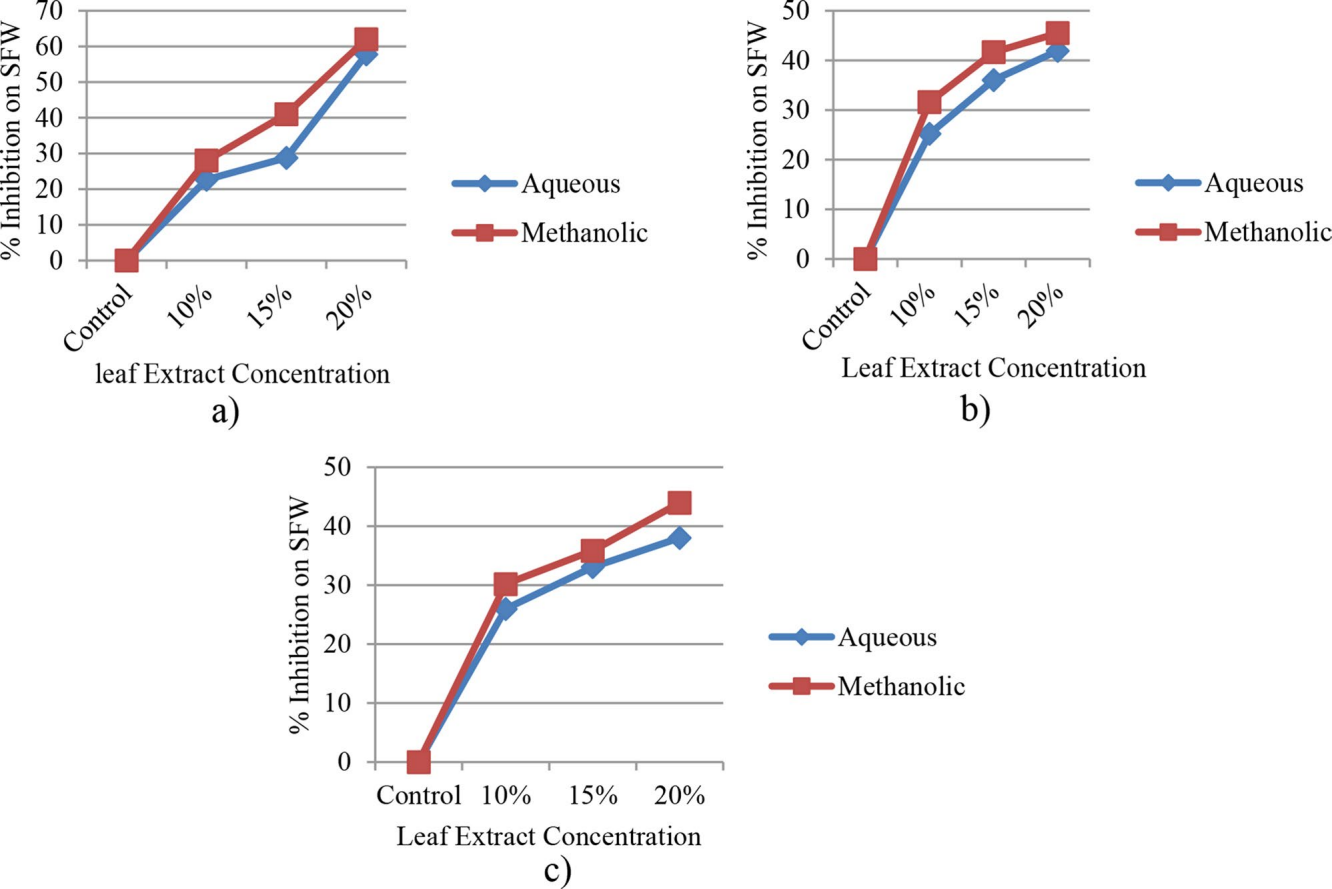
Percent inhibition with different levels of concentration of leaf extracts of *E. camaldulensis* on SFW of Assasa (**a**), Ude (**b**), and Mukiye (**c**) cultivars

The results revealed that in all treatment groups, both aqueous and methanolic leaf extracts of *E. camaldulensis* significantly (at *p* ≤ 0.05) decreased the weight of newly grown shoots in the three cultivars, in comparison to the control group (Fig. 6a-c). However, the most notable inhibitory impact was observed with the application of methanolic leaf extracts of *E. camaldulensis*. Khan et al. [52] also corroborated this finding, demonstrating that the shoot fresh weight in maize seedlings was reduced by the aqueous extract of *E. camaldulensis*.

#### Root fresh weight (RFW)

**Fig. 7:**
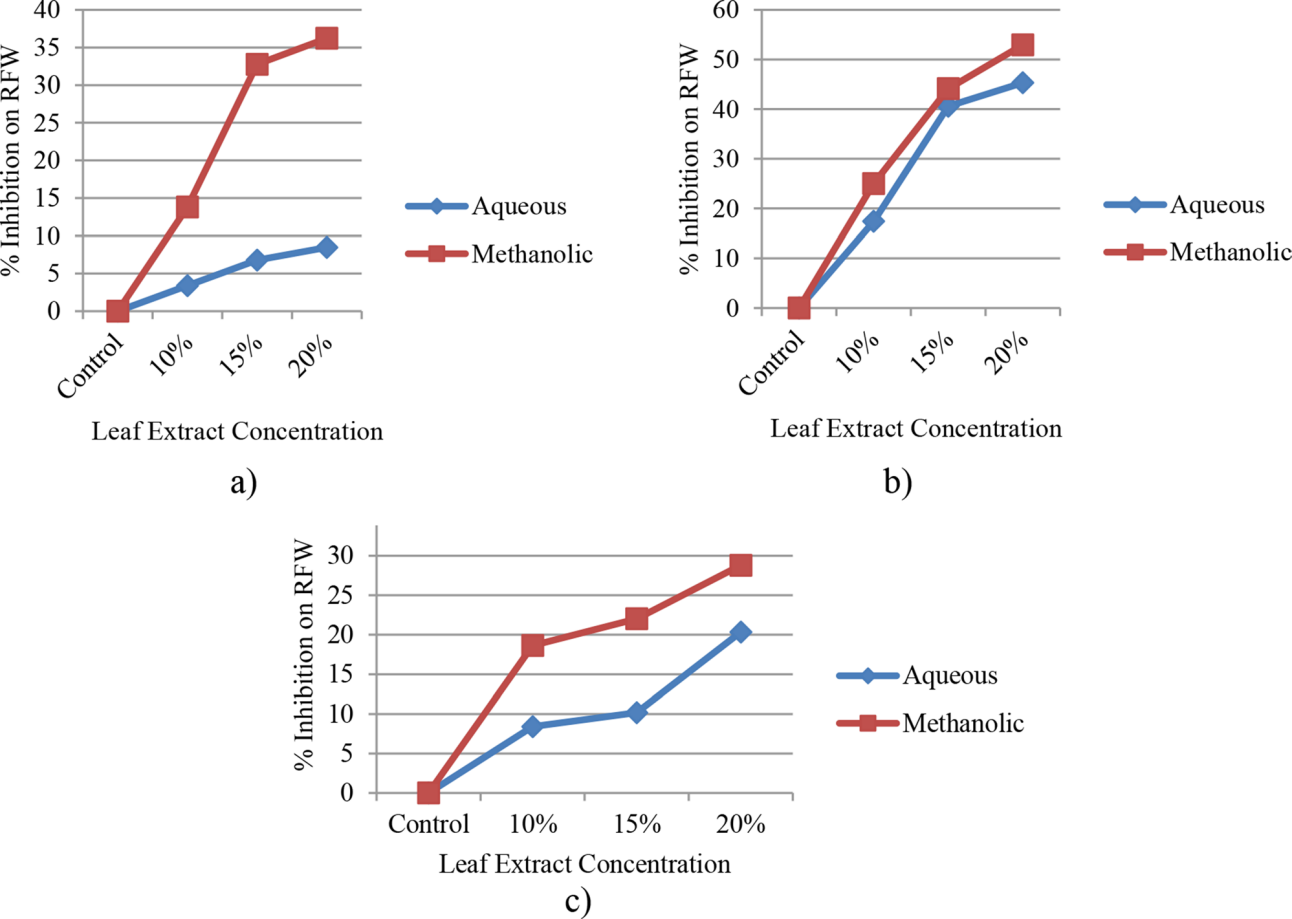
Percent inhibition with different levels of concentration of leaf extracts of *E. camaldulensis* on RFW of Assasa (**a**), Ude (**b**), and Mukiye (**c**) cultivars

The result indicated that all levels of treatment of the two leaf extracts obtained from *E. camaldulensis* markedly (at *p* ≤ 0.05) decreased the fresh weight of the roots when compared to the control group (Fig. 7a-c). It was discovered that the inhibition percentage at a concentration of 20% of the methanolic leaf extract was 36.21%, 52.4%, and 28.8% in the Assasa, Ude, and Mukiye cultivars, respectively. Conversely, the inhibition percentage at a concentration of 20% of the aqueous leaf extracts was 8.47%, 45.31%, and 20.34% in the Assasa, Ude, and Mukiye cultivars, respectively. The decline in fresh and dry weights could be attributed to the decrease in physiological processes such as photosynthesis [25]. The root fresh weight of the three cultivars was also significantly reduced at 20% concentration of both aqueous and methanolic leaf extracts. This is consistent with the report of Dawar et al. [67].

#### Yield fresh weight (YFW)

**Fig. 8:**
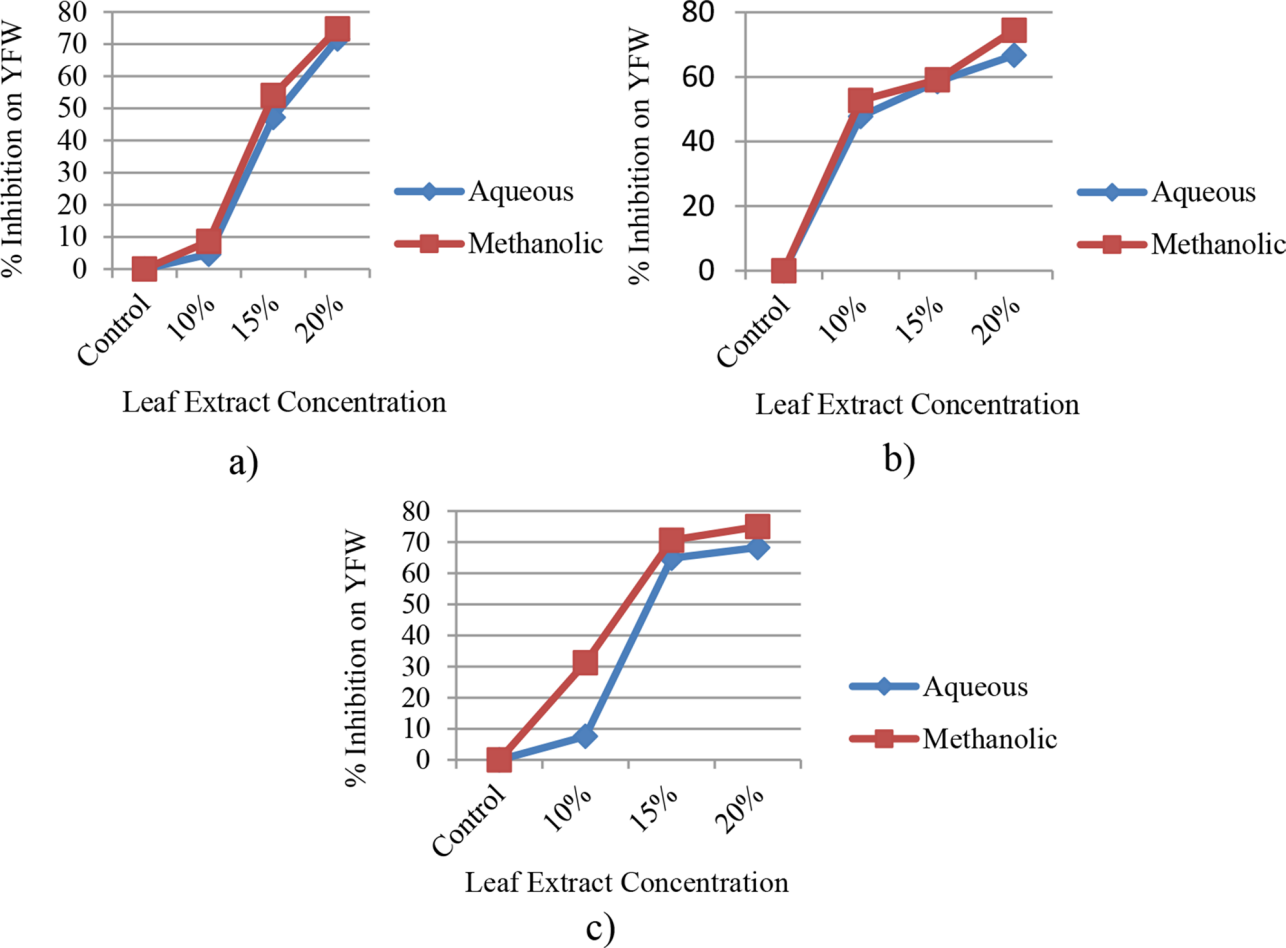
Percent inhibition with different levels of concentration of leaf extracts of *E. camaldulensis* on YFW of Assasa (**a**), Ude (**b**), and Mukiye (**c**) cultivars

The result showed that the yield of fresh weight for the three cultivars was considerably lower (*p* ≤ 0.05) at a concentration of 20% for both aqueous and methanolic leaf extracts, in comparison to the controls (Fig. 8a-c). At a higher concentration of methanolic leaf extract in Assasa, Ude, and Mukiye cultivars, the yield of fresh weight decreased by 74.71%, 74.45%, and 75%, respectively, whereas the percent reduction was 71.6%, 66.67%, and 68.28% in the same cultivars under the effect of aqueous extracts. Therefore, it can be deduced that the inhibitory effects of methanolic leaf extracts were greater than those of aqueous leaf extracts on the yield of fresh weight. This finding is in agreement with previous reports [62, 68, 69].

#### Shoot and root dry weight

**Fig. 9:**
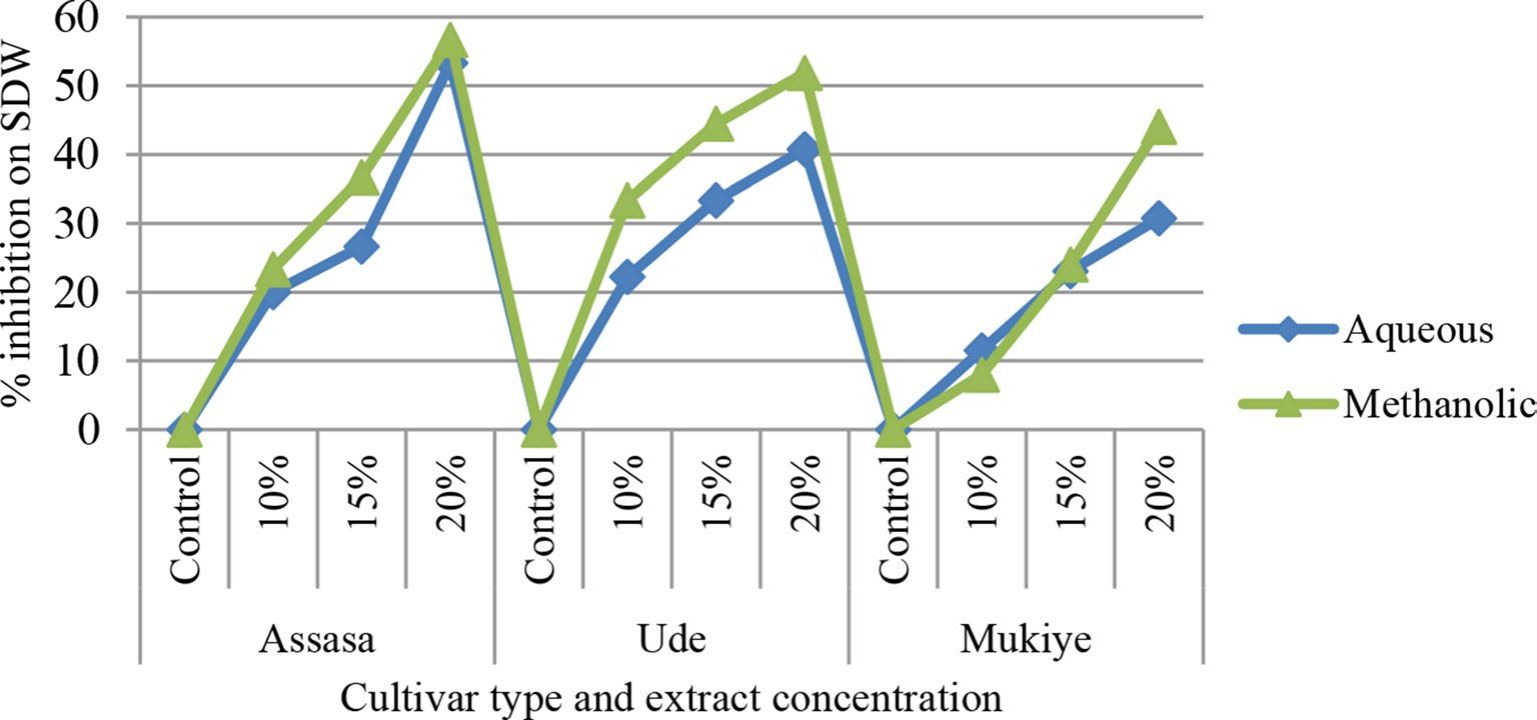
Percent inhibition of extract concentration on SDW of the three cultivars

**Fig. 10:**
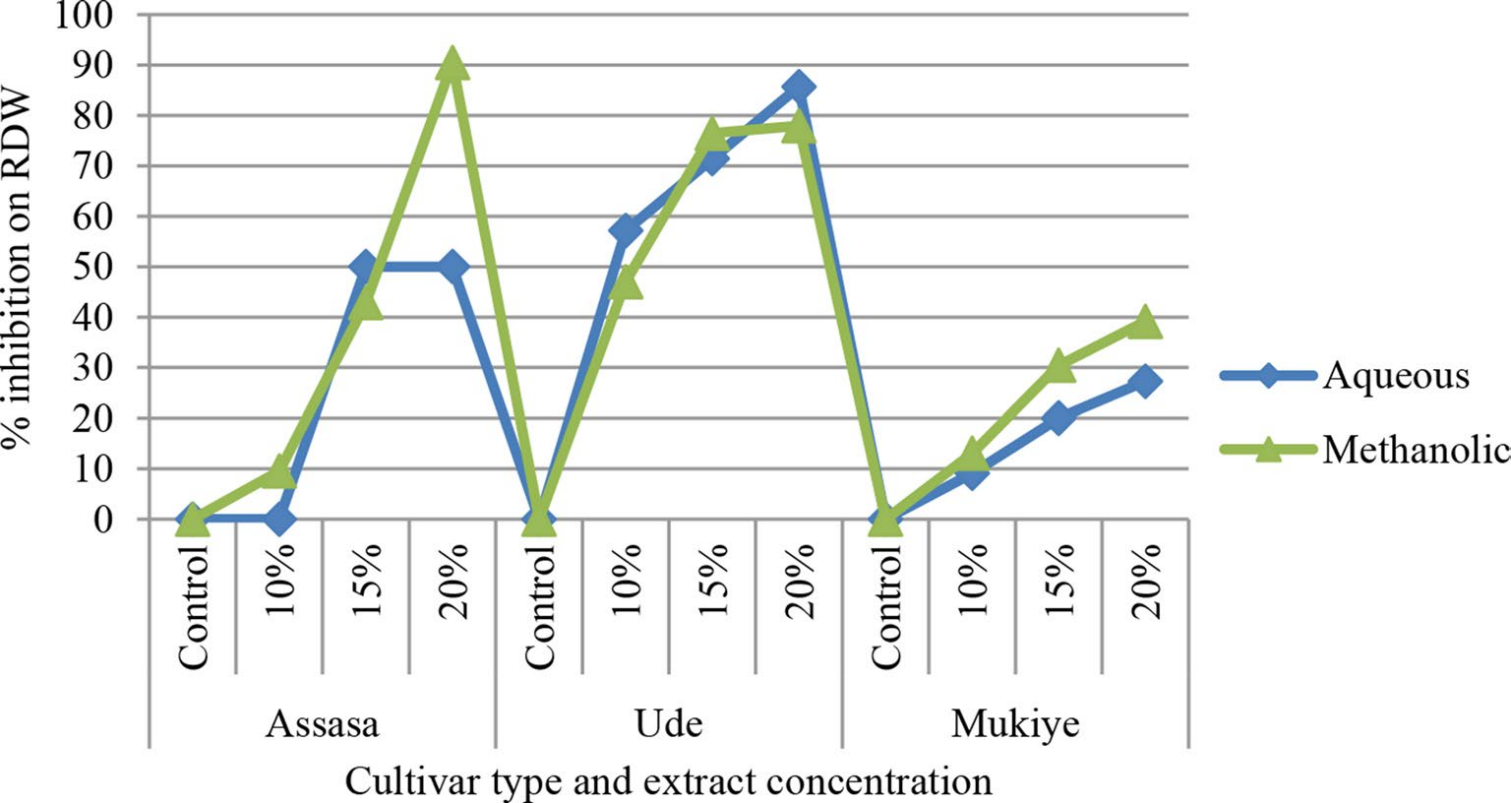
Percent inhibition of extract concentration on RDW of the three cultivars

**Fig. 11:**
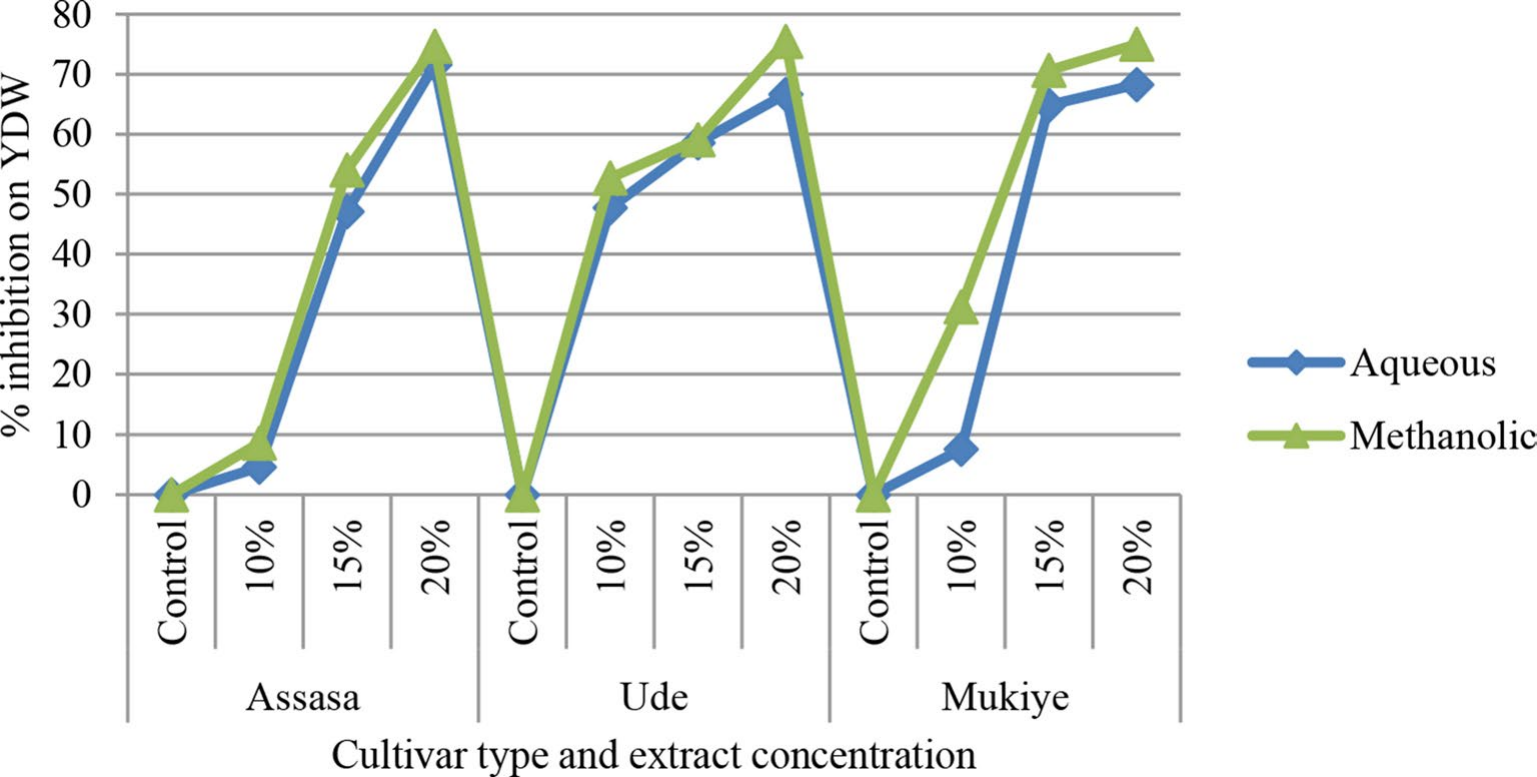
Percent inhibition of extract concentration of yield dry weight (YDW) of the three cultivars

The maximum decline in the average shoot dry weight of the three cultivars was observed at a 20% concentration of methanolic leaf extract obtained from *E. camaldulensis* treated pots in comparison to the control (Fig. 9). At higher concentrations of the extract, the percentage of inhibition was 53.33%, 40.74%, and 30.77% in aqueous leaf extract of Assasa, Ude, and Mukiye cultivars, respectively, whereas it was 56.67%, 51.85%, and 44% in methanolic leaf extract of *E. camaldulensis*. It has been discovered that all concentrations of aqueous and methanolic leaf extracts on the chosen cultivars lowered the dry matter production of wheat seedlings. This is in accordance with prior studies [50, 70–74]. The decrease may be due to stunted and reduced growth of seedlings [75]. Root dry weight (RDW) significantly declined at 20% concentration of both the aqueous and methanolic leaf extracts of *E. camaldulensis* (Fig. 10). Similar reports were obtained in the prior studies [76, 77]. Ghanuni et al. [78] also reported the effects of aqueous leaf extracts of *E. camaldulensis* on the dry weights of peanuts (*Arachis hypogaea* L.). Similarly, Dafaallah and EL-Towm [79] examined the effects of methanolic leaf extracts of *E. camaldulensis* on the shoot and root dry weights of different crops. It was found that the yield dry weight (YDW) was significantly reduced at a 20% concentration of both the aqueous and methanolic leaf extracts of *E. camaldulensis* (Fig. 11). This finding confirms the reports by Regu [80] and Hossain et al. [42].

## Interaction effects on the cultivars

The result of two-way ANOVA showed the interaction effect of cultivar type and aqueous leaf extract concentration on leaf area, shoot length, root length, shoot fresh weight, root fresh weight, shoot dry weight, root dry weight and yield fresh weight as shown in Tables 5 and 6. As the results showed, there was a statistically significant interaction between the effects of cultivar type and extract concentration on shoot length and root length of cultivars (*p* = 0.000, *p* = 0.001). However, there was a statistically insignificant interaction between the effects of cultivar type and extract concentration on fresh root weight and shoot dry weight among the three cultivars (*p* = 0.019, *p* = 0.049).

**Table 5:** ANOVA results of the effect of aqueous leaf extract of *E. camaldulensis* on some traits of the three wheat cultivars

Sources of variation	Mean squares								
	Df	LA	SL	RL	SFW	RFW	SDW	RDW	YFW
Cultivar type (CV)	3	20.300^ns^	151.879^**^	9.896^**^	71.108^**^	.584^ns^	.174^ns^	0.088^**^	42.958^**^
Extract Conc.(C)	2	409.029^**^	738.734^**^	10.303^**^	871.502^**^	4.139^**^	2.181^**^	0.094^**^	122.471^**^
CVXC	6	15.255^ns^	21.128^**^	1.248^**^	25.585^**^	1.220^**^	.097^ns^	0.043^**^	10.622^**^
Error	24	5.016	0.642	0.229	0.836	0.382	0.038	0.001	0.111

ns- not significant and **-significant at *p* ≤ 0.05, Df = Degree of freedom

**Table 6:** ANOVA results of the effect of methanolic leaf extract of *E. camaldulensis* on some traits of the three wheat cultivars

Sources of variation	Mean squares								
	Df	LA	SL	RL	SFW	RFW	SDW	RDW	YFW
Cultivar type (CV)	3	23.279^**^	162.444^**^	8.217^**^	73.647^**^	0.251^**^	0.252^**^	0.086^**^	33.023^**^
Extract Conc.(C)	2	504.590^**^	1176.523^**^	21.463^**^	1091.446^**^	10.715^**^	3.128^**^	0.123^**^	138.737^**^
CVXC	6	14.393^**^	24.722^**^	.204^ns^	19.643^**^	0.677^**^	0.142^**^	0.047^**^	9.024^**^
Error	24	2.683	0.782	0.261	1.050	0.072	0.010	0.000	0.097

ns- not significant and **-significant at *p* ≤ 0.05

## Correlation results in the three cultivars

Correlations between different traits among the aqueous leaf extracts of *E. camaldulensis* were made and pooled as shown in Table 7 for the three cultivars (at *p* ≤ 0.05 level). The result showed that the most strongly correlated parameters were shoot length and shoot fresh weight in the cultivars (*r* = 0.924). This indicates that an increase in shoot length increases shoot fresh weight in cultivars. On the other hand, the least correlated parameters were root length with number of leaves (*r* = 0.267) at the 0.05 level in cultivars. Correlations between various traits among the *E. camaldulensis* leaf methanolic extracts were also established and summarized as shown in Table 8. The result showed that the most strongly correlated parameters were shoot length and shoot fresh weight in the cultivars (*r* = 0.932). On the other hand, the least correlated parameters were the root dry weight with the number of leaves (*r* = 0.485) at the 0.01 level in the cultivars.

**Table 7:** Correlation between different traits under aqueous leaf extract in the three cultivars

	SL	RL	SFW	RFW	SDW	RDW	ST	NO.L	LA	YFW	YDW
SL	1	0.611^**^	0.924^**^	0.499^**^	0.786^**^	0.606^**^	0.635^**^	0.602^**^	0.755^**^	0.886^**^	0.860^**^
RL		1	0.683^**^	0.648^**^	0.754^**^	0.780^**^	0.287^**^	0.267^**^	0.639^**^	0.616^**^	0.605^**^
SFW			1	0.553^**^	0.863^**^	0.592^**^	0.733^**^	0.684^**^	0.831^**^	0.831^**^	0.808^**^
RFW				1	0.614^**^	0.574^**^	0.474^**^	0.438^**^	0.648^**^	0.473^**^	0.664^**^
SDW					1	0.458^**^	0.818^**^	0.781^**^	0.804^**^	0.727^**^	0.742^**^
RDW						1	0.330^*^	0.331^*^	0.685^**^	0.448^**^	0.695^**^
ST							1	0.801^**^	0.795^**^	0.641^**^	0.660^**^
NO.L								1	0.705^**^	0.510^**^	0.584^**^
LA									1	0.694^**^	0.810^**^
YFW										1	0.867^**^
YDW											1

** Correlation is significant at the 0.01 level (2-tailed)

* Correlation is significant at the 0.05 level (2-tailed)

**Table 8:** Correlation between different traits under methanolic leaf extract in the three cultivars

	SL	RL	SFW	RFW	SDW	RDW	ST	NO.L	LA	YFW	YDW
SL	1	0.921^**^	0.932^**^	0.896^**^	0.855^**^	0.642^**^	0.810^**^	0.696^**^	0.824^**^	0.898^**^	0.832^**^
RL		1	0.876^**^	0.845^**^	0.766^**^	0.454^**^	0.724^**^	0.625^**^	0.714^**^	0.887^**^	0.712^**^
SFW			1	0.885^**^	0.884^**^	0.652^**^	0.866^**^	0.764^**^	0.882^**^	0.888^**^	0.851^**^
RFW				1	0.809^**^	0.744^**^	0.778^**^	0.740^**^	0.866^**^	0.800^**^	0.847^**^
SDW					1	0.589^**^	0.929^**^	0.812^**^	0.908^**^	0.782^**^	0.820^**^
RDW						1	0.533^**^	0.485^**^	0.730^**^	0.537^**^	0.760^**^
ST							1	0.882^**^	0.900^**^	0.787^**^	0.793^**^
NO.L								1	0.804^**^	0.600^**^	0.708^**^
LA									1	0.762^**^	0.906^**^
YFW										1	0.814^**^
YDW											1

** Correlation is significant at the 0.01 level (2-tailed)

## Conclusion

The results show that both aqueous and methanolic leaf extracts of Eucalyptus camaldulensis have allelopathic effects on the morphological, physiological, and yield parameters of three Ethiopian wheat cultivars. However, the methanolic extracts exhibited stronger inhibitory effects than aqueous extracts, significantly reducing shoot and root lengths, leaf numbers, and stem thickness, thereby hampering plant growth. The extracts also negatively affected relative water content, chlorophyll content, and chlorophyll fluorescence, resulted in decreased fresh and dry biomass in the wheat cultivars. However, there are a few restrictions. Because the experiment was carried out in controlled pot experiment, it might not accurately reflect field settings where soil type, moisture content, and pests all play a role. Moreover, the concentrations used may not perfectly represent those occurring in natural environments, which restrict the generalizability of the findings. The study’s emphasis on *T. durum* cultivars might limit its relevance to other wheat varieties. Future research should involve field trials to validate these findings across different environmental conditions and examine the effects of *E. camaldulensis* extracts on a wider range of wheat crops.

## Data Availability

The datasets during and/or analyzed during the current study are available from the corresponding author on reasonable request.
